# Prevalence and Determinants of Fall-Related Injuries among Older Adults in Ecuador

**DOI:** 10.1155/2014/863473

**Published:** 2014-10-02

**Authors:** Carlos H. Orces

**Affiliations:** Department of Medicine, Laredo Medical Center, 1700 East Saunders, Laredo, TX 78041, USA

## Abstract

*Objectives*. To estimate the prevalence and determinants of fall-related injuries in the previous year among adults aged 60 years or older in Ecuador. *Methods*. The prevalence of fall-related injuries was estimated using cross-sectional data from the first national survey of Health, Wellbeing, and Aging study. Logistic regression models were used to examine the associations between participants' demographic characteristics and fall-related injuries. *Results*. Of 5,227 participants with a mean age of 72.6 years, 11.4% (95% CI, 10.3%–12.7%) reported a fall-related injury in Ecuador, representing an estimated 136,000 adults aged 60 years or older. Fall-related injuries were more frequently reported among older adults residing in the most urbanized and populated provinces of the country. After controlling for potential confounders, self-reported race as Indigenous (OR 2.2; 95% CI, 2.11–2.31), drinking alcohol regularly (OR 2.54; 95% CI, 2.46–2.63), subjects with greater number of comorbid conditions (OR 2.03; 95% CI, 1.97–2.08), and urinary incontinence (OR 1.83; 95% CI, 1.79–1.87) were factors independently associated with increased odds of sustaining fall-related injuries. *Conclusions*. Fall-related injuries represent a considerable burden for older adults in Ecuador. The present findings may assist public health authorities to implement fall prevention programs among subjects at higher risk for this type of injury.

## 1. Introduction

Falls among older adults represent a major public health problem associated with increased morbidity, mortality, and health care costs [1, 2]. Approximately 10% of falls result in a major injury such as a fracture, serious soft tissue injury, or traumatic brain injury [3]. Previous studies have demonstrated that fall-related fractures treated in hospital emergency departments and hospitalizations for fall-related injuries are increasing among older adults in developed countries [4–6]. Overall, 44.2% of adults aged 65 years or older with fall-related fractures require hospitalization and hip fractures account for 48% of the hospitalizations for fall-related injuries among women [4, 6]. Although there is scarce data about the epidemiology of fall-related injuries among older Ecuadorians, a previous study suggested that the incidence of hip fracture increased annually by 3.9% in Ecuador between 1999 and 2008 [7]. Moreover, assuming that the average annual percentage change in hip fracture rates remains unchanged, the number of hip fractures in the country is projected to rise to 8,900 and 47,000 by the years 2030 and 2050, respectively [7]. Recently, a study using data from the first national survey of Health, Wellbeing, and Aging described that 37.4% of older Ecuadorians sustain a fall each year. Moreover, recurrent falls occurred in 23.0% of the subjects and among fallers 30.6% reported a fall-related injury [8].

In Ecuador, the proportion of adults aged 60 years or older was 8.6% in 2010 and it is projected to increase to 14.4% by 2030. Similarly, current life expectancy is 75.5 years and it may reach 79.2 years by 2030 [9]. These demographic changes alone may increase considerably the number of fall-related injuries among older adults. Therefore, the present study extends previous research and aims to estimate the prevalence of and characteristics associated with fall-related injuries among adults aged 60 years or older residing in the coastal and mountains regions of Ecuador.

## 2. Materials and Methods

The present population-based study was based on cross-sectional data from the first national survey of Health, Wellbeing, and Aging (Encuesta de Salud, Bienestar y Envejecimiento, SABE I), conducted by trained interviewers between June and August of 2009. The SABE I survey is a probability sample of households with at least one person aged 60 years or older residing in the Andes Mountains and coastal regions of Ecuador. In the primary sampling stage, a total of 317 sectors from the rural areas (<2,000 inhabitants) and 547 sectors from the urban areas of the country were selected from the 2001 population Census cartography. In the secondary sampling stage, 18 households within each sector were randomly selected based on the assumption that at least one person aged 60 years or older lives in 24% and 23% of the households in the coastal and Andes Mountains regions, respectively. Survey data, including operation manuals, are publicly available [10].

### 2.1. Fall-Related Injury Ascertainment

A fall-related injury was assessed by the following question: “Did you need medical attention after sustaining a fall?” Subjects who answered affirmatively to the question were considered to have developed a fall-related injury in the previous year.

### 2.2. Demographic and Health Characteristics

Age and sex were self-reported. The race of participants was classified according to the following question: “Do you consider yourself to be white, black, Mestizo, Mulatto, or Indigenous?” Body height in centimeters and weight in kilograms were measured and the body mass index was calculated (Kg/cm^2^). Participants were asked about their living status (alone versus living with others) and area of residence (urban versus rural). The average use of alcohol per week during the previous three months was classified as none, one day, or two or more days per week.

Self-reported general health was grouped as excellent to good or fair to poor. The number of comorbidities (0, 1, ≥2) was assessed by asking participants if they had been diagnosed by a physician with the following conditions: diabetes mellitus, chronic obstructive pulmonary disease, arthritis, stroke, coronary artery disease, or cancer. Urinary incontinence was defined as having involuntary incontinence of urine that occurred at least once during the previous year.

Cognitive status was evaluated by the abbreviated Mini Mental State Examination (MMSE). This modified MMSE was developed by Icaza and Albala to identify the MMSE questions that could best explain cognitive deterioration. The abbreviated MMSE was developed with nine variables instead of the 19 original MMSE variables. A cutoff point of 12 or less was defined to identify people with cognitive impairment [11]. The Geriatric Depression Scale was used to evaluate the presence of depressive symptoms. This 15-item scale has been validated in Spanish populations with a sensitivity of 81% and a specificity of 76%. Respondents with a score of 6 or more were considered to have symptoms of depression [12, 13]. The following activities of daily living (ADLs) were included in the present study: walking across a room, dressing, bathing, eating, getting in and out of bed, and using the toilet. Those participants who needed help or were unable to perform one or more of the ADLs were considered functionally impaired. Physical activity was evaluated by the question “Do you regularly exercise such as jogging, dance, or perform rigorous physical activity at least three times weekly for the past year?” Subjects who responded affirmatively were considered to engage in regular physical exercise.

Grip strength was evaluated using a standard hand-held dynamometer. Participants used their dominant hand and the average result of two trials was reported in Kg/sec. The chair stand test was used to assess lower-limb muscle strength. This test was considered successfully completed if participants were able to stand up five times from a chair with their arms folded within 60 seconds [14]. The results of the muscle strength measures were grouped into quartiles to examine the association between grip strength and lower-limb muscle strength and fall-related injuries. Balance was evaluated by the single leg stance test. Subjects who were able to stand in one foot for 10 seconds completed successfully the test.

### 2.3. Statistical Analysis

Categorical variables were compared using the chi-squared test. Those variables statistically significant (*P* value < 0.05) in the univariate analyses were entered into a multivariate regression model adjusted for age, gender, and body mass index to evaluate the independent associations between fall-related injuries and demographic and health characteristics of the participants. Results of the logistic regression model are presented as odds ratios (OR) with their 95% confidence intervals (95% CI). To compare the geographic distribution of this injury across the country, the age-specific proportions of fall-related injuries by provinces were age-adjusted by the direct method using the 2010 Census population of Ecuador as the standard. All analyses were weighted to account for the multistage sampling design of the SABE I survey. Statistical analyses were performed using SPSS, version 17 software (SPSS Inc., Chicago, IL).

## 3. Results

**Figure 1:**
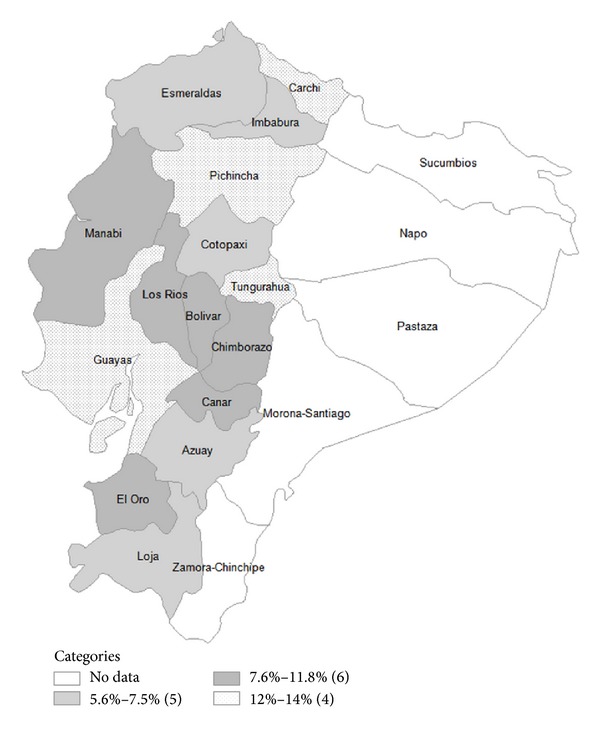
Fall-related injury prevalence rates by provinces in Ecuador.

Of 5,227 participants with a mean age of 72.6 years (8.9 years), 11.4% (95% CI, 10.3%–12.7%) reported a fall-related injury in the previous year, representing an estimated 136,000 adults aged 60 years and older in Ecuador. As shown in Figure 1, the prevalence of fall-related injuries varied across regions of the country. After age adjustment, higher fall-related injury rates were predominantly found among subjects residing in the provinces of Guayas and Pichincha, which are the most populated and urbanized provinces of the country.

As shown in Table 1, fall-related injury rates were considerably higher among Indigenous, those living alone, older adults who drink alcohol regularly, and participants with cognitive impairment and symptoms of depression. Moreover, subjects with symptoms of urinary incontinence or greater number of chronic comorbidities reported more frequently fall-related injuries as compared to those who did not. Of relevance, among subjects who completed the physical performance tests, fall-related injury rates progressively increased as the muscle strength decreased in both the grip-strength and chair stand tests.

As shown in Table 2, the results of the multivariate model indicate that after adjusting for age, sex, and BMI, Indigenous older adults, regular use of alcohol, self-reported health as fair to poor, having two or more chronic comorbidities, and symptoms of urinary incontinence were characteristics significantly associated with increased fall-related injury prevalence in Ecuador. Moreover, among subjects who completed the physical performance tests, those with the best scores on the grip strength test, chair-stand test, and single leg stance had 25%, 20%, and 13% lower risk of sustaining fall-related injuries as compared with subjects who performed worse on these tests, respectively.

## 4. Discussion

The results of the present study indicate that 11.4% of community-dwelling adults aged 60 years or older sustain a fall-related injury each year in Ecuador. In general, fall-related injury rates varied across the country. However, these injuries occurred predominantly among residents from the most populated and urbanized provinces of the country. Overall, the geographic distribution of fall-related injuries among older adults in Ecuador contrasts with results from a recent investigation that demonstrated higher fall prevalence rates among older subjects residing in the rural Andes Mountains of the country [8]. Similarly, previous studies have demonstrated higher incidence of fall-related injury rates among subjects residing in rural areas [15, 16].

Of relevance, a marked racial disparity in fall-related injuries was seen among older Ecuadorians. For instance, self-reported race as Indigenous was a variable associated with 1.8-fold increased odds of sustaining fall-related injuries as compared with the White. The reasons for higher fall-related injury prevalence rates among this minority ethnic group in Ecuador are unknown. However, high risk occupations among Indigenous people such as farming and construction may partly explain the present findings.

The higher prevalence of fall-related injuries with increasing age and among women found in the present study is consistent with results from previous investigations [17, 18]. Previous studies also have reported that gender differences in fall-related injuries may be attributed to 2- to 3-fold higher fractures rates among women [4, 17–19]. Moreover, gender differences in fall-related injuries have been related to higher prevalence of osteoporosis, frailty, muscle strength, and willingness to seek medical attention among women [20–23].

Self-reported health status as fair to poor and greater number of comorbidities were variables associated with increased odds of sustaining fall-related injuries among older adults in Ecuador. The present findings are consistent with results from a recent study reporting that fair to poor health among older adults was associated with 3-fold increased risk of sustaining fall-related injuries among people aged 85 years or older in the previous 3 months [24]. Moreover, the number of comorbidities has been associated with increased risk for fall-related injuries [25, 26]. For instance, Tinetti et al. demonstrated that community dwelling persons aged 72 years or older with at least two chronic conditions had 2-fold higher odds of sustaining fall-related injuries, which is similar to the present findings [26].

Interestingly, subjects who reported symptoms of urinary incontinence had 1.7-fold higher odds of sustaining fall-related injuries. In Ecuador, urinary incontinence among older adults also was previously found to be an independent factor associated with increased odds of sustaining a fall in the previous year [8]. Falls related to urine incontinence are generally thought to result from loss of balance when rushing to the toilet. However, it is unclear whether incontinence is a primary cause of falls or it is simply a marker of physical frailty [27].

In Ecuador, fall-related injury rates were 10% higher among older adults who took part in rigorous physical activity at least three times weekly as compared with those who did not. In contrast with the present results, an earlier study reported that vigorous physical activity decreased fall-related fracture risk among older adults with no limitations in ADL [28]. Likewise, Cummings et al. demonstrated that women who walked for exercise had a 30% lower risk of hip fracture as compared with those who did not [29]. The reason for the increased fall-related injury risk associated with intense exercise found in the present study is uncertain. However, consistent with the present findings, a recent cross-sectional study among community-dwelling adults aged 50 years and older showed that the likelihood of falling increased by 5% for each 100 metabolic expenditure (MET-min/week) of vigorous-intensity physical activity [29]. Apparently, changes in standing balance among older adults following moderate physical exercise may be a predisposing factor for fall-related injuries [30].

Regular use of alcohol was a potentially modifiable factor associated with increased prevalence of fall-related injuries among older Ecuadorians. In fact, compared with nondrinkers, older adults who self-reported drinking on average 2 or more days per week during the previous 3 months had 2.5-fold higher odds of sustaining fall-related injuries. Similarly, a previous cross-sectional study among older adults in Cataluña, Spain, demonstrated that subjects who drink alcohol heavily had 1.2-fold higher odds of reporting a fall-related injury during the previous year [31]. On the contrary, a recent analysis from the Behavioral Risk Factor Surveillance System Survey found no statistically significant association between consumption of alcohol and fall-related injuries among older adults aged 85 years or older [24]. A possible explanation for these contradictory results may be related to differences in survey definitions regarding alcohol consumption among older adults.

Participants who scored in the highest quartile on the muscle strength measures had considerably lower odds of sustaining fall-related injuries as compared to those in the lowest quartile. The present findings are consistent with results of a systematic review and meta-analysis, which demonstrated that lower extremity weakness is a clinically significant risk factor for falls and fall-related injuries [32]. Previously, lower extremity weakness evaluated by the chair stand test also was found to be associated with higher prevalence of falls among older adult in Ecuador [8]. Likewise, weak grip strength has been reported to be a significant predictor for recurrent falls and nonsyncopal fall-related injuries among community-dwelling older adults [33, 34].

Several limitations must be mentioned in interpreting the present results. First, participants used self-reports of sociodemographic characteristics, medical diagnoses, and ADL's limitations, which may be a source of recall bias. Second, the SABE I survey did not collect data on specific types of fall-related injury, such as fracture, contusion, abrasion, and laceration. Likewise, other variables associated with increased risk for fall-related injuries such as orthostatic hypotension, bone mineral density, or use of psychotropic drugs were not investigated. Third, the present results may be only generalized to older adults residing in the coastal and Andes Mountains regions of the country. However, older adults from the Amazon region and the Galapagos Islands represented only 3.3% of the population aged 60 years or older in Ecuador [35]. Despite these limitations, this study is the first to estimate the prevalence of fall-related injuries and to examine characteristics associated with this type of injury among older adults in Ecuador.

In conclusion, fall-related injuries represent a considerable burden for older adults in Ecuador. The present findings may assist public health authorities to implement fall prevention programs among subjects at higher risk for this type of injury.

## Figures and Tables

**Table 1:** Prevalence of fall-related injuries among older adults in Ecuador.

Characteristics	Number of subjects	% (95% CI)
Gender		
Women	2,766	13.9 (12.2–15.8)
Men	2,466	8.5 (7.2–10.0)
Age groups, yrs		
60–69	2,441	9.5 (8.0–11.3)
70–79	1,645	12.7 (10.7–15.2)
≥80	926	14.4 (11.7–17.7)
Area of residence		
Rural	2,354	9.8 (8.4–11.4)
Urban	2,878	12.3 (10.7–14.0)
Race		
Indian	530	15.0 (11.6–19.2)
Black	169	9.4 (5.6–15.5)
Mestizo	3,349	11.7 (11.2–13.4)
Mulatto	179	8.7 (4.8–15.0)
White	670	8.9 (6.7–11.8)
Living arrangements		
Alone	547	15.4 (11.1–20.9)
Accompanied	4,684	11.0 (9.8–12.2)
BMI (Kg/m^2^)		
Underweight	163	10.9 (6.4–17.9)
Normal weight	2,045	10.4 (8.7–12.4)
Overweight	1,892	10.6 (8.8–12.6)
Obese	843	12.0 (9.2–15.5)
Alcohol use		
None	4,167	12.2 (10.9–13.6)
1 day	939	7.2 (5.5–9.4)
≥2 days	123	19.3 (9.9–34.2)
Regular physical activity		
No	3,573	12.1 (10.7–13.6)
Yes	1,657	10.1 (8.1–12.5)
GDS ≥ 6		
No	2,633	9.6 (8.1–11.4)
Yes	1,153	14.5 (12.2–17.1)
Cognitive impairment		
No	3,719	9.6 (8.4–10.9)
Yes	1,079	15.9 (13.0–19.2)
ADL's limitations		
No	3,771	9.9 (8.6–11.3)
Yes	1,454	15.6 (13.3–18.1)
Self-reported health		
Excellent to good	1,197	8.1 (6.2–10.5)
Fair to poor	4,027	12.6 (11.2–14.1)
Urinary incontinence		
No	4,041	9.5 (8.4–10.7)
Yes	1,169	17.7 (14.7–21.1)
Comorbidities		
0	2,442	7.8 (6.5–9.4)
1	1,763	13.3 (11.3–15.6)
≥2	860	17.2 (14.1–20.8)
Grip strength (Kg/sec)		
Q1 (1 to 15)	1,301	16.5 (14.0–19.3)
Q2 (16 to 20)	1,205	10.7 (8.5–13.4)
Q3 (21 to 27)	1,198	8.7 (6.9–11.0)
Q4 (28 to 97)	1,214	8.1 (6.1–10.7)
Chair stand test (sec)		
Q1 (4 to 9)	1,187	9.8 (7.6–12.5)
Q2 (10 to 11)	1,041	8.4 (6.3–11.1)
Q3 (12 to 14)	1,073	7.7 (6.0–9.8)
Q4 (≥15)	919	14.0 (11.1–17.7)
Single leg stance (sec)		
0 to 9 sec	2,147	10.1 (8.7–11.9)
10 sec	1,947	8.0 (6.5–9.8)

GDS: Geriatric Depression Scale; BMI: body mass index.

**Table 2:** Characteristics of participants associated with fall-related injuries.

	Unadjusted OR (95% CI)	Adjusted OR (95% CI)^a^
Age groups, yrs		
60–69	1.00	1.00
70–79	1.39 (1.37–1.40)	1.37 (1.36–1.39)
≥80	1.60 (1.58–1.62)	1.63 (1.60–1.65)
Gender		
Men	1.00	1.00
Women	1.74 (1.72–1.76)	1.79 (1.77–1.82)
BMI (Kg/m^2^)		
Underweight	1.00	1.00
Normal	0.95 (0.92–0.99)	0.94 (0.91–0.98)
Overweight	0.97 (0.93–1.00)	0.96 (0.92–0.99)
Obesity	1.11 (1.07–1.16)	1.02 (0.98–1.06)
Area of residence		
Rural	1.00	1.00
Urban	1.29 (1.27–1.30)	1.02 (1.02-1.02)
Race		
Indian	1.79 (1.75–1.84)	1.87 (1.82–1.92)
Black	1.06 (1.02–1.10)	1.35 (1.29–1.41)
Mestizo	1.35 (1.33–1.38)	1.49 (1.46–1.52)
Mulatto	0.96 (0.93–1.00)	0.79 (0.76–0.83)
White	1.00	1.00
Living arrangements		
Alone	1.47 (1.45–1.50)	1.35 (1.33–1.37)
Accompanied	1.00	1.00
Alcohol use		
None	1.00	1.00
1 day	0.56 (0.55-0.56)	0.80 (0.78–0.81)
≥2 days	1.71 (1.66–1.77)	2.54 (2.46–2.63)
Regular physical activity		
No	1.00	1.00
Yes	0.81 (0.80–0.82)	1.10 (1.09–1.12)
GDS ≥ 6		
No	1.00	1.00
Yes	1.58 (1.56–1.61)	1.37 (1.35–1.39)
Cognitive impairment		
No	1.00	1.00
Yes	1.78 (1.75–1.80)	1.49 (1.46–1.51)
ADL's limitations		
No	1.00	1.00
Yes	1.68 (1.66–1.70)	1.33 (0.64–0.68)
Self-reported health		
Excellent to good	1.00	1.00
Fair to poor	1.63 (1.61–1.66)	1.60 (1.57–1.62)
Urinary incontinence		
No	1.00	1.00
Yes	2.05 (2.03–2.08)	1.77 (1.75–1.79)
Comorbidities		
0	1.00	1.00
1	1.81 (1.78–1.83)	1.52 (1.49–1.54)
≥2	2.44 (2.40–2.48)	2.22 (2.19–2.26)
Grip strength (Kg/sec)		
Q1 (1 to 15)	1.00	1.00
Q2 (16 to 20)	0.60 (0.59–0.61)	0.65 (0.64–0.66)
Q3 (21 to 27)	0.48 (0.47–0.49)	0.61 (0.60–0.62)
Q4 (28 to 97)	0.44 (0.43–0.45)	0.75 (0.74–0.77)
Chair stand test (sec)		
Q1 (4 to 9)	0.66 (0.65–0.67)	0.80 (0.78–0.81)
Q2 (10 to 11)	0.56 (0.55–0.57)	0.61 (0.60–0.63)
Q3 (12 to 14)	0.50 (0.49–0.51)	0.53 (0.52–0.54)
Q4 (≥15)	1.00	1.00
Single leg stance (sec)		
0 to 9 sec	1.00	1.00
10 sec	0.76 (0.75–0.77)	0.87 (0.86–0.88)

GDS: Geriatric Depression Scale; ^a^adjusted for age, sex, and body mass index.
